# Bone Mineral Density Reporting Underestimates Fracture Risk in Ontario

**DOI:** 10.4236/health.2015.75067

**Published:** 2015-05

**Authors:** Joanna E. M. Sale, Earl Bogoch, Lynn Meadows, Monique Gignac, Lucy Frankel, Taucha Inrig, Dorcas Beaton, Ravi Jain

**Affiliations:** 1 Musculoskeletal Health and Outcomes Research, Li Ka Shing Knowledge Institute, St. Michael’s Hospital, Toronto, Canada; 2 Institute of Health Policy, Management & Evaluation, University of Toronto, Toronto, Canada; 3 Mobility Program, St. Michael’s Hospital, Toronto, Canada; 4 Department of Surgery, University of Toronto, Toronto, Canada; 5 Community Health Sciences, University of Calgary, Calgary, Canada; 6 Toronto Western Research Institute, University Health Network, Toronto, Canada; 7 Dalla Lana School of Public Health, University of Toronto, Toronto, Canada; 8 Osteoporosis Canada, Toronto, Canada

**Keywords:** Fracture Risk, Bone Densitometry Reports, Fragility Fracture, Ontario

## Abstract

**Objective:**

Analysis of clinical documents such as bone mineral density (BMD) reports is an important component of program evaluation because it can provide insights into the accuracy of assessment of fracture risk communicated to patients and practitioners. Our objective was to compare fracture risk calculations from BMD test reports to those based on the 2010 Canadian guidelines.

**Methods:**

We retrieved BMD reports from fragility fracture patients screened through a community hospital fracture clinic participating in Ontario’s Fracture Clinic Screening Program. Fracture risk was determined according to the 2010 Canadian guidelines using age, sex, and T-score at the femoral neck, in addition to three clinical factors. Three researchers classified patients’ fracture risk until consensus was achieved.

**Results:**

We retrieved reports for 17 patients from nine different BMD clinics in the Greater Toronto Area. Each patient had a different primary care physician and all BMD tests were conducted after the 2010 Canadian guidelines were published. The fracture risk of 10 patients was misclassified with 9 of the 10 reports underestimating fracture risk. Nine reports acknowledged that the prevalence of a fragility fracture raised the risk category by one level but only four of these reports acknowledged that the patient had, or may have sustained, a fragility fracture. When we raised fracture risk by one level according to these reports, eight patients were still misclassified. Fracture risk in the majority of these patients remained underestimated. Inconsistent classification was found in the majority of cases where reports came from the same clinic. Four reports described risk levels for two different types of risk.

**Conclusions:**

More than half of patients received BMD reports which underestimated fracture risk. Bone health management recommendations based on falsely low fracture risk are likely to be sub-optimal.

## 1. Introduction

The 2010 Clinical Practice Guidelines for the Diagnosis and Management of Osteoporosis in Canada [1] specify that all fragility fracture patients over the age of 50 undergo bone mineral density (BMD) testing and be assessed for future fracture risk. Ontario’s Fracture Clinic Screening Program was created in high volume fracture clinics in 2005 to address previous recommendations for fragility fracture patients [2], and to ensure that Canadian clinical practice guidelines are followed. Systematic reviews have shown modest improvements in BMD testing [3]–[5] and treatment initiation [3]–[6] after patients have been screened through Fracture Liaison Services [7] [8], such as the Fracture Clinic Screening Program. In order to explain the gaps in treatment initiation *after* BMD testing, studies have examined patients’ interpretation of fracture risk [9] [10]; however, less attention has been given to the BMD reports themselves. One study conducted prior to the release of the 2010 Canadian guidelines showed that BMD reports underestimated fracture risk [11].

Analysis of clinical documents such as BMD reports is an important component of program evaluation [12] because it can provide insights into the accuracy of assessment of fracture risk communicated to patients and practitioners. The purpose of this study was to compare fracture risk calculations from BMD test reports in patients screened through the Fracture Clinic Screening Program to those based on the 2010 Canadian guidelines which refered to either the revised CAROC [13] or Canadian FRAX [14] tool for fracture risk calculation.

## 2. Methods

This audit sampled data from BMD reports of patients screened through a community hospital fracture clinic participating in the Fracture Clinic Screening Program. This site screens approximately 250 fragility fracture patients annually. Recruitment of patients over 17 months was conducted at a pace to accommodate qualitative data collection about barriers to care from the patient perspective [15]. All patients had been assessed by an osteoporosis screening coordinator, educated about bone health, and advised to follow up with their primary care physician for a BMD test and appropriate treatment. Each patient’s primary care physician referred him or her to a BMD clinic and made treatment decisions. Patients were identified by the screening coordinator as meeting our eligibility criteria if they were 50+ years old, had sustained a fragility fracture, were not on antiresorptive or other bone-active medication at the time of fracture, and had followed up with a BMD test as recommended. A research coordinator interviewed patients to confirm the location of the current fracture and to ascertain whether they had sustained a previous fragility fracture. This study was approved by the Research Ethics Board of St. Michael’s Hospital in Toronto.

An in-depth examination of the BMD reports was conducted by three researchers. Based on the 2010 guidelines [1], fracture risk was determined by age, sex, and T-Score at the femoral neck, in addition to three clinical factors (prevalent fracture, prevalent vertebral or hip fracture, history of fragility fracture). The guidelines classify fracture risk as either “moderate” or “high” in patients 50+ who have sustained a fragility fracture [1]. The three researchers independently classified patients’ fracture risk according to the guidelines and then met to discuss their classifications until consensus was achieved.

## 3. Results

We retrieved BMD reports from 17 fragility fracture patients who were screened through the fracture clinic; the remaining patients from the qualitative study (n = 8) did not undergo a BMD test as recommended. Each of the 17 patients had a different primary care physician and we confirmed that all BMD tests were conducted after the 2010 Canadian guidelines [1] were published. Nine different BMD clinics in Ontario, one with 4 locations, generated the 17 BMD reports. These clinics were dispersed across an area of 360 square kilometres, and thus allowed us to examine reporting mechanisms across a variety of clinics in the Greater Toronto Area.

BMD reports classified the fracture risk levels of 7 patients correctly but in 5 of the 7 reports, how risk status was calculated was unclear. For example, no reason was given for fracture risk status even though the reported T Score was not sufficient for determining the patient as “moderate risk” (e.g. ID23), or the report claimed that the lowest T Score was indicative of “high” risk when the T Score on its own was actually indicative of “moderate” risk (e.g. ID2, ID6). In 2 of the 7 patients, the reason for classification was clear in that the report acknowledged the patient had a history of fracture (although not definitive that it was a *fragility* fracture) (ID16) or that the patient had sustained a fragility fracture (ID22). The 2010 guidelines account for additional clinical factors (e.g. corticosteroid use) but we did not have access to patients’ charts so were unable to include these additional clinical factors in our analysis unless they were present in the BMD reports. Five reports indicated that the patient had no history of corticosteroid use (ID2; ID7; ID19; ID20; ID25) but only one acknowledged that corticosteroid use would raise fracture risk by one level (ID7).

Fracture risk levels in 10 patients were misclassified. Of the 10 misclassified cases, the BMD reports underestimated fracture risk in 9 patients and possibly overestimated fracture risk in one patient. Therefore, across all reports, fracture risk was underestimated in 9/17, or 53%, of cases. For example, despite the Canadian guidelines specifying that all patients with a fracture are to be classified no less than “moderate” risk, 6 of the 10 reports classified patients as “low” risk.

Nine reports acknowledged that the prevalence of a fragility fracture placed patients at increased risk, or raised patients’ risk category by one level, but only four of these reports mentioned that the patient had a history of fracture, had sustained a recent or previous fracture, or had sustained a *fragility* fracture (ID16; ID18; ID21; ID22). When we raised risk categories by one level according to the BMD reports recommending this practice, 8 of the 17 patients were still misclassified. Fracture risk in the majority of these patients (5/8) was still underestimated (equivalent to 29% of all patients). In one case, risk was underestimated by two levels (ID11). In other words, the BMD report classified the patient as low risk and our research team classified the patient as high risk according to the guidelines. Conversely, another BMD report included the instruction to raise fracture risk by one category when the patient was already specified as “high” risk in the report (ID2).

Five of the nine clinics represented by our sample generated 2+ reports each. We compared the reports within these five clinics. Within 4 of the 5 clinics, the reports were both consistent and inconsistent compared with our risk calculations, meaning that different reporting and interpretation mechanisms were used within the same clinics.

Seven of the reports referred to the original CAROC guidelines [16], two appeared to refer to the current CAROC [13] (citing them as “Osteoporosis Canada 2010 Guidelines for the Assessment of Fracture Risk (2011)” or the “Recommendations for Bone Mineral Density Reporting in Canada (2010)”), two referred to the “current guidelines of the International Society of Clinical Densitometry and Osteoporosis Canada” (no date specified), and the remaining reports did not say or were unclear about how fracture risk was calculated. In general, the revised CAROC guidelines are more conservative than the previous guidelines in that they tend to designate fewer patients as “high” risk for future fracture. Four reports described two different types of risk levels. According to these four reports, fracture risk status, as well as risk levels to qualify for the Ontario Health Insurance Plan (OHIP) benefits was described. OHIP is a program for reimbursement and does not set guidelines. For example, two patients were classified as low fracture risk but elsewhere in the report, they were classified as high risk for OHIP (ID9, ID12) and two were classified as moderate fracture risk but later described as high risk for OHIP (ID14, ID22). Patients who are high risk for OHIP are permitted to have funded annual BMD tests in the province of Ontario, whereas patients with lower risk levels are generally funded for BMD tests at intervals of 3–5 years.

## 4. Discussion

Based on our study, BMD reports underestimated fracture risk in 53% of patients who had a BMD test. Fracture risk was underestimated in many cases because the classification process failed to account for clinical factors that contribute to fracture risk such as the presence of a prevalent fragility fracture. In fact, only four of the 17 reports included documentation that the patient had presented with a previous fracture or fragility fracture. Our results are consistent with those of a recent Canadian study [11]. However, that study was conducted prior to the publication of the 2010 guidelines and the authors excluded patients screened through the Fracture Clinic Screening Program. Differences in reporting were also found *within* clinics represented in our sample. Further, we determined that there was heterogeneity in the tools used to determine risk in the BMD reports examined, with only two reports appearing to rely on the current Canadian guidelines. To add to the confusion, four reports described risk levels for two different types of risk.

While it is encouraging that some reports qualified risk levels by providing instructions for risk levels to be increased by one category if the patient had sustained a fragility fracture, we do not know if all primary care physicians were familiar with the current guidelines’ determination of fracture risk and we do not believe all primary care physicians followed the instructions in the BMD reports to increase risk levels appropriately. It was recently demonstrated that some primary care physicians in Ontario reported computing risk assessments in their practice even when provided with assessments on BMD reports [17], however, several authors [15] [18] have shown that health care providers often do not consider fractures from standing height or less [19] to be fragility fractures. We suggest that BMD reports need to consistently account for clinical factors such as fragility fracture status so that health care providers do not have to interpret the results further. We also suggest that the BMD reports be standardized so that all fracture risk assessments are based on the same fracture risk algorithm.

One limitation of our study was that we did not have the requisitions for the BMD tests so cannot comment on whether these forms included clinical information such as fragility fracture status of the patient. It was also not possible for us to determine whether the primary care physician relayed information to the imaging physician about other clinical risk factors. Further, we do not know who in the clinics was responsible for fracture risk assessment. In order for the reports to accurately reflect risk, there must be accurate flow of information either from the referring primary care physician to the imaging physician, and/or the provision for the patient to be questioned regarding relevant clinical information. Further, there must be appropriate recognition and use of this clinical information by the imaging physician.

While our sample was small, we retrieved 100% of the BMD reports that were generated. These reports represented 17 different primary care physicians as well as nine different clinics in Ontario, one with four locations, that were widely dispersed across the Greater Toronto Area.

## 5. Conclusion

More than half of BMD reports underestimated fracture risk in patients who underwent BMD testing from a community hospital fracture clinic participating in Ontario’s Fracture Clinic Screening Program. The most important purpose of post-fracture screening is to identify the highest risk individuals for future hip and vertebral fractures, and to initiate preventive measures including pharmacotherapy where indicated to reduce that risk. If BMD reports underestimate the fracture risk of patients and accurate risk information is not provided to patients and to the clinicians responsible for ordering the tests, these patients may fail to receive the indicated interventions. Thus, if family physicians and bone health specialists rely on BMD reports alone to make treatment decisions, screening programs will fail to prevent future hip and vertebral fractures. This compounds the already complex challenges of risk reduction as illustrated in earlier research, such as disruptions in the circle of care [20] and patients’ understanding of risk [21].
